# Diversity of Acyl Homoserine Lactone Molecules in Anaerobic Membrane Bioreactors Treating Sewage at Psychrophilic Temperatures

**DOI:** 10.3390/membranes10110320

**Published:** 2020-10-30

**Authors:** Shamas Tabraiz, Burhan Shamurad, Evangelos Petropoulos, Alex Charlton, Obaidullah Mohiudin, Mohammad Danish Khan, Emeka Ekwenna, Paul Sallis

**Affiliations:** 1 School of Engineering, Newcastle University, Newcastle Upon Tyne NE1 7RU, UK; b.a.s.shamurad@newcastle.ac.uk (B.S.); vagpetrop@gmail.com (E.P.); O.Mohiuddin2@newcastle.ac.uk (O.M.); danishkhan2547upss@gmail.com (M.D.K.); paul.sallis@newcastle.ac.uk (P.S.); 2 School of Natural and Environmental Sciences, Newcastle University, Newcastle Upon Tyne NE1 7RU, UK; alex.charlton@ncl.ac.uk; 3 Department of Engineering, Durham University, Durham DH1 3LE, UK; emeka.b.ekwenna@durham.ac.uk

**Keywords:** quorum sensing, acyl homoserine lactone, anaerobic membrane bioreactor

## Abstract

This study explores the types of acyl homoserine lactone (AHL) and their concentrations in different compartments of different conventional anaerobic bioreactors: (i) an upflow anaerobic membrane bioreactor (UAnMBR, biofilm/mixed liquor (sludge)); (ii) an anaerobic membrane bioreactor (AnMBR, biofilm/mixed liquor (sludge)); and (iii) an upflow sludge blanket (UASB, sludge only), all operating at 15 °C. Ten types of the AHL, namely C4-HSL, 3-oxo-C4-HSL, C6-HSL, 3-oxo-C6-HSL, C8-HSL, 3-oxo-C8-HSL, C10-HSL, 3-oxo-C10-HSL, C12-HSL, and 3-oxo-C12-HSL, which were investigated in this study, were found in UAnMBR and UASB, whilst only six of them (C4-HSL, 3-oxo-C4-HSL, C8-HSL, C10-HSL, 3-oxo-C10-HSL, and C12-HSL) were found in AnMBR. Concentrations of total AHL were generally higher in the biofilm than the sludge for both membrane bioreactors trialed. C10-HSL was the predominant AHL found in all reactors (biofilm and sludge) followed by C4-HSL and C8-HSL. Overall, the UAnMBR biofilm and sludge had 10-fold higher concentrations of AHL compared to the AnMBR. C10-HSL was only correlated with bacteria (*p* < 0.05), whilst other types of AHL were correlated with both bacteria and archaea. This study improves our understanding of AHL-mediated Quorum Sensing (QS) in the biofilms/sludge of UAnMBR and AnMBR, and provides new information that could contribute to the development of quorum quenching anti-fouling strategies in such systems.

## 1. Introduction

Anaerobic treatment is considered a more suitable option for wastewater treatment (focusing on the organic fraction) due to the low energy requirement, the potential for resource recovery (methane production and/or other intermediates), and the lower sludge production [1] compared to conventional aerobic treatment that has been used historically. Recently, to improve anaerobic treatment, membranes have been introduced internally and/or externally to anaerobic bioreactors [2,3,4,5]. However, membrane fouling, particularly through biofilm formation, is a perpetual challenge that hampers the widespread use of anaerobic membrane bioreactors (AnMBR) for wastewater treatment applications. Typically, the energy required for gas sparging to control the membrane fouling in AnMBR could be up to 86% of the total energy requirement [6]. Thus, the AnMBR technology could have a greater appeal if the energy requirement for mixing/gas sparging could be substantially reduced [7]. Hence, there is a need for improvements related to membrane operation and fouling mitigation, especially for substrates that are low in organic matter, and consequently have low potential for energy recovery (i.e., domestic wastewater). 

Bacteria initiate biofilm formation through a mechanism of excretion and sensing diffusible molecules (autoinducers), which is known as quorum sensing (QS). Numerous Gram-negative bacteria have been reported previously to express QS activity via acyl homoserine lactones (AHL) [8,9,10,11]. The structure of AHL consists of a homoserine lactone (HSL) ring attached to an acyl chain length of between C4 and C18; the acyl chain comes with or without ‘‘oxo’’ or ‘‘hydroxyl’’ groups at the C3 position [12,13,14,15]. 

Biofilm formation in aerobic MBR has been previously linked with AHL [16,17,18]. The increased AHL concentration has been found to correlate with extracellular polymeric substances (EPS), which subsequently increase fouling [19,20,21]. Since biofouling is connected with the presence of AHL in aerobic MBR [22,23,24,25], AHL have been successfully targeted to control biofilm formation and reduce biofouling in aerobic systems. Among the AHL, most studies on aerobic MBR have targeted the C8-HSL molecule, and consider it a plausible cause of fouling [16,26,27]. However, the presence of AHL in anaerobic engineered systems has been reported rarely and not investigated in detail. The formation of anaerobic granules in an UASB reactor has also been linked to AHL [28]. The majority of studies used synthetic wastewater anaerobic granules to study AHL, whilst to date only one study reported the AHL status in actual industrial wastewater using anaerobic granular sludge [29]. Different types of AHL molecules have been investigated in different processes of anaerobic digestion [30]. A recent study also investigated the exogenous addition of AHL in anaerobic granules and reported this enhanced the performance of granules in terms of removal efficiencies for organic carbon [31]. 

Although several studies report the presence of AHL in UASB reactors, more information is required with regard to the types and quantities of AHL in anaerobic membrane bioreactors treating real wastewater. Therefore, this study investigated the status of AHL in the sludge and biofilm of conventional AnMBR and upflow anaerobic membrane bioreactor (UAnMBR), as well as in the sludge of a UASB reactor, all treating real sewage. Specifically, the work focuses on the relationship between the microbial community profile and the AHL detected in these membrane/sludge-based anaerobic systems, especially when they operate under extreme conditions (i.e., low temperatures). Low-temperature anaerobic treatment (with and without a membrane) has been has been investigated previously, but no information was provided about the AHL status [32,33]. Only a few studies have reported the AHL status in anaerobic systems (specifically UASB) operated at mesophilic conditions (30–37 °C) [28,29,30]. However, the current study explores the AHL status in conventional AnMBR and UAnMBR as well as in the sludge of a UASB reactor treating sewage at a psychrophilic temperature (15 °C). 

## 2. Materials and Methods

### 2.1. Experimental Setup

All the bioreactors had been previously operated continuously for 1 year prior to sampling. Experimentation took place in the Environmental Engineering Laboratory, School of Engineering, Newcastle University, UK. The AnMBR, UAnMBR, and UASB were run continuously for 200 days during the current experiment. The bioreactors had originally been inoculated with cold adapted biomass acclimated to “cold” naturally due to its origin (Lake Geneva, Swizerland and Savalbard, Norway) and further acclimated to UV-sterilized wastewater as a substrate through numerous feed batches over a 12-month period [34]. The influent was primary settled sewage collected from the Northumbrian Water Tudhoe Mill wastewater treatment plant, Durham, UK, which was collected monthly and stored at 4 °C. For this trial, unsterilized substrate was fed to the reactors. No pH adjustment was made to the substrate, as it ranged between 6.7 and 7.2. The operational parameters of the bioreactors and wastewater characteristics are given in Table 1. The membrane flux of AnMBR and UAnMBR was estimated by measuring the volume of the effluent on a daily basis (collected in 24 h). The membranes were backwashed daily for 30 min with permeate effluent. 

### 2.2. AHL Molecules Investigated in the Study

The types of acyl homoserine lactones (AHL) used in the current study were selected after an extensive literature review. Only AHL which had been reported in activated sludge, or in the strain cultures isolated from it, were selected [35,36]; N-butanoyl-L-homoserine lactone (C4-HSL), N-3-oxo-butanoyl-L-homoserine lactone (3-oxo-C4-HSL), N-hexanoyl-L-homoserine lactone (C6-HSL), N-3-oxo-hexanoyl-L-homoserine lactone (3-oxo-C6-HSL), N-octonoyl-L-homoserine lactone (C8-HSL), N-3-oxo-octonoyl-L-homoserine lactone (3-oxo-C8-HSL), N-decanoyl-L-homoserine lactone (C10-HSL), N-3-oxo-decanoyl-L-homoserine lactone (3-oxo-C10-HSL), N-dodecanoyl-L-homoserine lactone (C12-HSL), and N-3-oxo-dodecanoyl-L-homoserine lactone (3-oxo-C12-HSL). These AHL were purchased from Chemodex, St. Gallen, Switzerland. 

### 2.3. Sludge and Biofilm Collection and AHL Extraction

Sludge and biofilm were collected from AnMBR and UAnMBR, while only sludge was collected from UASB. Two samples of biofilm and sludge were collected on the 170th and 180th day of experimentation. The sample collection time was selected when steady-state conditions were evident from the operational parameters after a prolonged period (≈2 months). 

AHL from the biofilm and sludge were extracted using a modified Lade et al. (2014) method [35]. Briefly, the used membrane from the AnMBR and UAnMBR was disconnected and placed in a tube containing 50 mL phosphate buffer solution (PBS) solution. The tube was closed tightly and shaken for 2 min by hand. The suspension of the biofilm (BF) and mixed liquor sludge (S), 50 mL each, were centrifuged at 10,000× *g* for 10 min. The supernatant was filtered through a 0.2 μm cellulose acetate filter. The filtrate was mixed with an equal volume of ethyl acetate and shaken at 180 rpm for 2 h. The top organic layer was collected and dried via nitrogen gas (99.9%) purging. The dried residue was dissolved in 0.5 mL solution of acetonitrile and formic acid (0.1%) [23]. 

### 2.4. AHL Identification and Quantification

Standard stock solutions of each AHL at 1 mg mL^−1^ were prepared in acetonitrile. AHL standards of different concentrations—0.015 µM, 0.03125 µM, 0.0625 µM, 0.125 µM, 0.25 µM, 0.5 µM and 1.0 µM—were made by diluting the stock solution in acetonitrile/0.1% formic acid solution in appropriate proportions to prepare the standard curve. An ultra-performance liquid chromatograph coupled with triple quadrupole mass spectrometer (UPLC-MS/MS) (Waters, Xevo TQ-XS, Milford, MA, USA) was used to identify and quantify the AHL. The column used for the analysis was an Acquity BEH C18 (2.1 × 100 mm; 1.7 µm Particle Size) (Waters, UK). The temperature of the column was kept at 20 °C. Two mobile phases were used: (a) water + formic acid (0.1%) and (b) acetonitrile + formic acid (0.1%). The solvent gradients (time: % B) used were (0.0: 30), (5.0: 30), (12.0: 90), (12.5: 90), (15: 30), (17: 95), (18: 30), and (20: 30). 

Standards and AHL extracts from the biofilm and mixed liquor sludge were injected at the rate of 0.25 mL min^−1^. The MS settings were as follows: ionization mode, positive; ionization source, electrospray ionization; capillary voltage, 3.0 kV; cone voltage, 30 V; source offset, 50 V; desolvation gas glow, 650 L h^−1^; desolvation temperature, 350 °C; cone gas flow, 150 L h^−1^; collision gas flow, 0.2 mL min^−1^; nebulizer gas pressure, 7.0 bar and collision energy, 2 eV. Column effluent was detected using the multiple reaction monitoring approach. The specific liquid chromatography time, appearance of precursor’s ions (m/z) and two transition ions, and the relative intensity of the two transition ions was used as a reference (m/z; 102, m/z; 74). A standard curve was prepared from the transition ions with the highest intensity. 

### 2.5. Influent/Effluent Quality Analysis

The chemical oxygen demand (COD) in the influent and effluent were measured using standard methods, APHA (2006). The COD removal efficiency was estimated using the formula below. The mixed liquor suspended solids (MLSS) content of the biomass in the bioreactors was quantified gravimetrically [37].

(1) $$Removal\;efficiency=\frac{CODin-CODout}{CODin}\times 100$$

### 2.6. EPS Extraction, Proteins, and Polysaccharides Measurement

The scraped biofilm was suspended in phosphate buffer solution (PBS) to make a 10 mL sample volume. The biofilm and PBS suspension were shaken well by hand to disperse the biofilm thoroughly. Sludge and biofilm suspension (10 mL) were centrifuged for 5 min at 6000 × *g* (4 °C). The supernatant was collected, and a 0.2 μm cellulose acetate filter (Millipore, Merk UK) was used to filter the suspended particles. The content of the proteins (PN) and polysaccharides (PS) in the solution represented the soluble EPS/soluble microbial product (SMP). The sludge and biofilm sample pellets were resuspended in 10 mL PBS and sonicated for 2 min using ultrasonic cleaner (9USC-TH, VWR, Bristol, UK). The suspension was placed in a shaker (KS400i, IKA, Oxford, UK) at 150 rpm for 10 min and centrifuged at 8000× *g* for 10 min. The harvested supernatant was filtered, and the PN and PS present in the filtrate were denoted loosely bound EPS (LB-EPS). The pellets in tubes were re-suspended in 10 mL PBS and re-sonicated for 3 min. Subsequently, the sludge was exposed to sonication for 3 min. In each tube (10 mL), 2 g of hydrated CER (cation exchange resin) (Dowex^®^ Marathon^®^ C sodium form, Sigma-Aldrich, Kent, UK) was added in the suspension. Then, the solution was centrifuged at 12,000 × *g* (30 min) and the content of PS and PN in the supernatant was defined as tightly bound EPS (TB-EPS) [38,39,40].

### 2.7. Molecular Microbial Analysis

DNA Extraction: Due to the nature of the inoculum (soils and sediments), a phenol extraction method was used with minor modification for the DNA extraction [41]. Briefly, the biomass obtained from the biofilm and mixed liquor sludge was centrifuged at 4000× *g* for 30 min, and the supernatant was removed. The CTAB buffer (0.5 mL), phenol:chloroform:isoamyl alcohol (25:24:1) (Sigma Aldrich) solution, was added, and the pellets were resuspended. The mixture was transferred to the lysing matrix-E tubes (Sigma, UK). Afterwards, the tubes were placed in a ribolyzer (30 sec, 4.0 m sec^−1^) followed by centrifugation for 5 min at 16,000× *g* and 5 °C. The supernatant was transferred to the phase lock gel^®^, green tubes (VWR, UK). Then, 0.5 mL of phenol/isoamyl alcohol (24:1) was added to the phase lock gel tubes, mixed well, and centrifuged at 16,000× *g* and 5 °C for 5 min. To remove the phenol completely and produce a high-quality supernatant, the phase lock gel (green tube) step was repeated twice. Supernatant was transferred to a 2 mL Eppendorf tube, precipitated by adding two volume of 30% PEG (6000) (Sigma Aldrich, UK) solution, and mixed well. The sample was placed at 5 °C overnight. The mixture was centrifuged for 20 min at 16000× *g* and 5 °C. The supernatant was discarded, and 1 mL of ethanol (70%, ice-cold, filtered) was added. The solution was centrifuged at 16,000× *g* and 5 °C for 20 min. The supernatant was discarded and tubes were spun down for 1–2 s. The remaining ethanol was removed, and tubes were dried at 55 °C for 1–2 min. The DNA eluted with ultrapure DPEC water (Thermofisher, Dartford, UK). The quantity and quality of the extracted DNA was checked by Nano-drop (Thermofisher, UK). The DNA was saved at −80 °C for further use.

Sequencing: Polymerase chain reaction (PCR) of the extracted DNA was carried out using the pair of universal reverse; primer 806R (GGACTACHVGGGTWTCTAAT) and the forward primer 515F (GTGCCAGCMGCCGCGGTAA), targeting the V4 region of 16S rRNA gene [42,43]. The GoTaq^®^ Hot Start master mix (Thermo Fisher Scientific, UK) was used for the PCR with the following conditions: initial denaturation (94 °C, 3 min), denaturation 35 cycles (94 °C, 45 s), annealing (50 °C, 30 s), extension (70 °C, 90 s). The quality control was carried out by agarose gel, library preparation including adaptors tagging, and an equal concentration of all samples were mixed and cleaned up. After amplification, sequencing was carried out at the prepared samples using the Illumina MiSeq platform (NU-OMICS, Northumbria University, Newcastle Upon Tyne, UK) to identify the 16S rDNA amplicon.

The raw data obtained from the illumina MisSeq were de-multiplexed and filtered using dada2 for quality control [44] within the QIIME2 analysis pipeline, https://qiime.org [45]. To get the close-reference amplicon sequence variant (ASV), VSEARCH was used by plug in the cluster-features-closed-reference in QIIME2 [46]. Adding to it, the SILVA119 database was used to produce the table with frequencies of the taxonomically assigned representative sequences.

### 2.8. Data Visualization

Non-metric multidimensional scaling (NMDS) plots of the microbial communities in biofilm and sludge samples from all bioreactors was performed using Phyloseq Package [47]. The relative abundance bar chart, the canonical correspondence analysis (CCA) between AHL and the microbial community, and a heat map of correlation between proteins/polysaccharides and AHL concentration were prepared using Microbiome Package [48] in R (R Studio v3.6.3) following the procedures described by Shamurad et al. [49,50]. For co-occurrence analysis, a correlation matrix was developed by calculating all possible pairwise Spearman correlations among the AHL and microbial community (genus level) of UASB, UAnMBR, and AnMBR (*n* = 5). A correlation between AHL and microbial community was considered if the Spearman’s correlation coefficient was ≥ 0.8 and the *p* value was ≤ 0.05. To reduce the chances of obtaining false-positive results, the *p* values were adjusted with a multiple testing correction using the Benjamini–Hochberg method [51]. The pairwise correlations of the AHL and the bacterial/archaea genus formed their co-occurrence networks. Network analyses were performed in R environment and the microbial communities were further visualized and explored to identify their topological properties (i.e., clustering coefficient, shortest average path length and modularity) in Gephi [52].

## 3. Results

### 3.1. AHL Types and Concentrations

**Figure 1:**
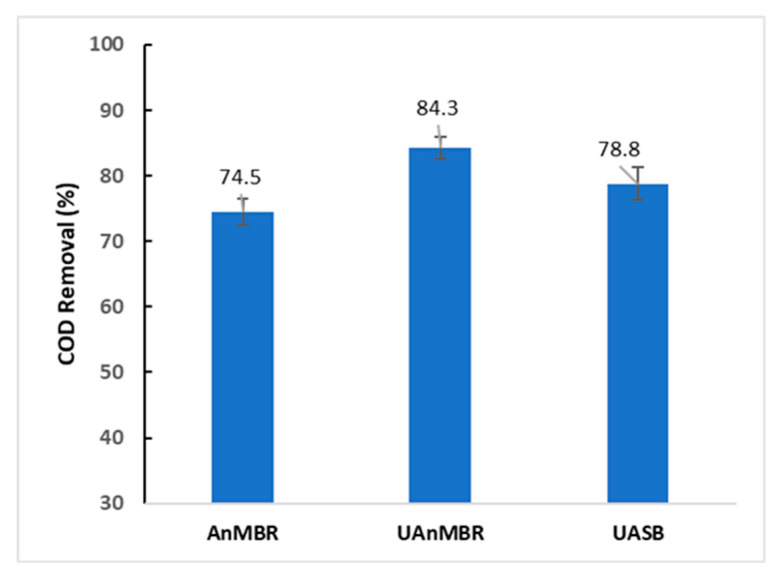
Chemical oxygen demand (COD) percentage removal efficacies monitored in the last two months of operation to access the steady-state conditions of all the bioreactors. Sample were taken after every 5 days (*n* = 12). The error bar represents standard deviation.

**Figure 2:**
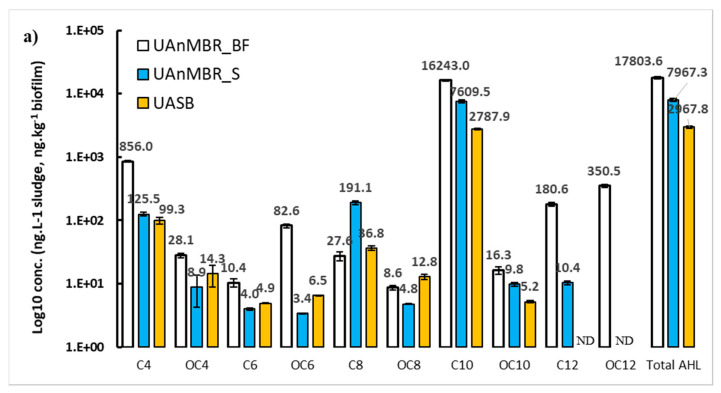

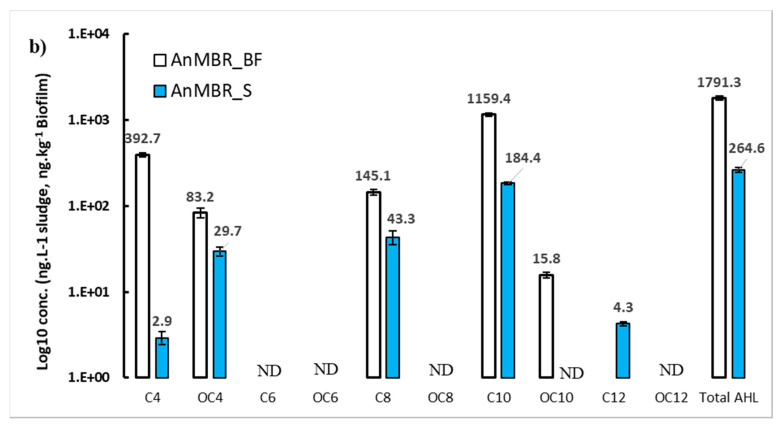
Acyl homoserine lactone (AHL) concentrations in the biofilm (ng kg^−1^) and sludge (mg L^−1^) of (**a**) UAnMBR and UASB (sludge only), (**b**) AnMBR (error bars show standard deviation of replicates; *n* = 2); the *y*-axis is a log scale. AHL abbreviations are; C4: C4-HSL; C6: C6-HSL; C8: C8-HSL; C10: C10-HSL; C12: C12-HSL; OC4: 3-oxo-C4-HSL; OC6: 3-oxo-C6-HSL; OC8: 3-oxo-C8-HSL; OC10: 3-oxo-C10-HSL; OC12: 3-oxo-C12-HSL. The abbreviation of ND; not detected.

Ten types of AHL were evaluated in the biofilm and sludge of the two conventional anaerobic membrane bio-reactor types: (i) UAnMBR, (ii) AnMBR, and in the (iii) sludge of a UASB reactor. The samples for AHL analysis were taken when the bioreactor reached a pseudo-steady state condition. This condition was determined from the COD removal efficiencies of the bioreactors, which were consistent through the final two months of operation (Figure 1). The concentration and types of these AHL varied with reactor type (Figure 2a,b). Biofilms in both membrane systems had a higher concentration of AHL than the sludge. The C10-HSL was the most abundant AHL at all treatments (present in both sludge and biofilm for all setups), followed by C4-HSL and C8-HSL.

Overall, 10 different AHL were found in the UAnMBR biofilm, showing greater diversity than AnMBR biofilm where only six AHL were detected. The AHL:C6-HSL, 3-O-oxo-C6-HSL, 3-oxo-C8-HSL, and 3-oxo-C12-HSL were not detected in the AnMBR biofilm. In addition to the lower AHL diversity, the concentrations of the total AHL in the AnMBR biofilm were also lower (10-fold) compared to the UAnMBR biofilm. Briefly, the concentrations of C4-HSL and C10-HSL in UAnMBR were 2.2- and 14-fold higher than the those in AnMBR, respectively, while the concentration of 3-oxo-C10-HSL was found to be similar in both biofilms. In contrast, the concentrations of 3-oxo-C4-HSL and C8-HSL were 3- and 5-fold higher in AnMBR biofilm than in the UAnMBR biofilm. The sludge of the UAnMBR had a higher concentration of all AHL than UASB sludge except for 3-oxo-C4-HSL, C6-HSL, 3-oxo-C6-HSL, and 3-oxo-C8-HSL.

The total AHL concentrations in the UAnMBR sludge were 30- and 2.7-fold higher than those found in the AnMBR and UASB sludge, respectively. Similarly, the concentration of C10-HSL in the sludge of UAnMBR was 41- and 2.7-fold higher than that in the AnMBR and UASB sludge, respectively. The C4-HSL concentration in the AnMBR sludge was 30- to 40-fold lower than that in the UASB and UAnMBR sludge, while C8-HSL in the UAnMBR sludge was 4- to 5-fold higher than that in the UASB and UAnMBR sludge.

### 3.2. Protein and Polysaccharide Correlations with AHL

**Figure 3:**
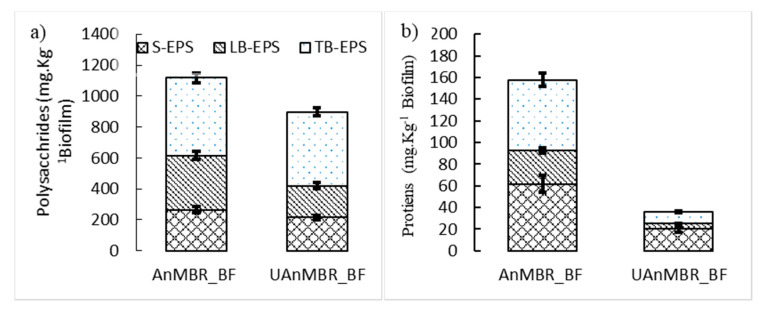

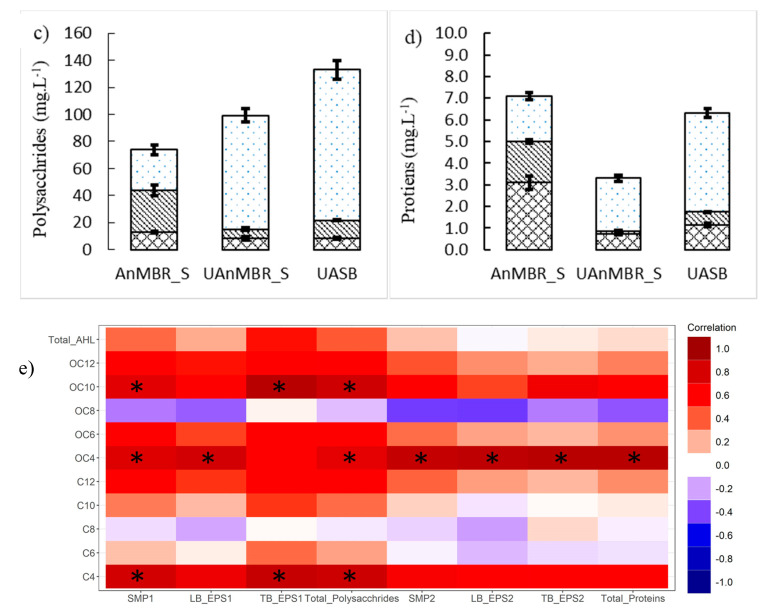
(**a**) Polysaccharides in soluble extracellular polymeric substance (S_EPS), loosely bound extracellular polymeric substance (LB_EPS), and tightly bound extracellular polymeric substance (TB_EPS) in AnMBR_BF and UAnMBR_BF; (**b**) proteins in S_EPS, LB_EPS, and TB_EPS in AnMBR_BF and UAnMBR_BF; (**c**) polysaccharides in S-EPS, LB-EPS, and TB-EPS in AnMBR_S, UAnMBR_S, and UASB; (**d**) proteins in S_EPS, LB_EPS, and TB_EPS in AnMBR_S, UAnMBR_S, and UASB. The concentrations of the proteins and polysaccharides in the biofilms are reported in mg kg^−1^, while in the sludge, they are reported in mg L^−1^ (error bars represent standard deviation of replicates; *n* = 2). (**e**) Pearson correlation between proteins, polysaccharides, and AHL present in the biofilm and sludge of the AnMBR, UAnMBR, and UASB. S_EPS1: polysaccharides in soluble extracellular polymeric substances; LB.EPS1: polysaccharides in loosely bound extracellular polymeric substances; TB.EPS1: polysaccharides in tightly bound extracellular polymeric substances; S.EPS2: proteins in soluble extracellular polymeric substances; LB.EPS2: proteins in loosely bound extracellular polymeric substances; TB.EPS2: proteins in tightly bound extracellular polymeric substances. The asterisk (*) indicates *p* < 0.05. AHL abbreviations are; C4: C4-HSL; C6: C6-HSL; C8: C8-HSL; C10: C10-HSL; C12: C12-HSL; OC4: 3-oxo-C4-HSL; OC6: 3-oxo-C6-HSL; OC8: 3-oxo-C8-HSL; OC10: 3-oxo-C10-HSL; OC12: 3-oxo-C12-HSL.

The concentration of polysaccharides was found to be higher than that of proteins in both the biofilms and the sludge samples for all three reactors. The biofilms of the AnMBR and UAnMBR had higher concentrations of polysaccharides and proteins as compared to the sludge for all reactors. Interestingly, despite the relatively low organic loading rates, the concentrations of both polysaccharides and proteins were higher in the AnMBR biofilm than those found in the UAnMBR biofilm. On the contrary, the polysaccharide concentration in the AnMBR sludge was lower than that in the UAnMBR sludge, whilst the polysaccharide concentration was the highest in the UASB sludge (Figure 3a–d). Three AHL (C4-HSL, 3-oxo-C4-HSL, and 3-oxo-C10-HSL) were correlated significantly (*p* < 0.05) with the concentration of polysaccharides and proteins (Figure 3e).

### 3.3. Microbial Community in AnMBR, UAnMBR, and UASB

**Figure 4:**
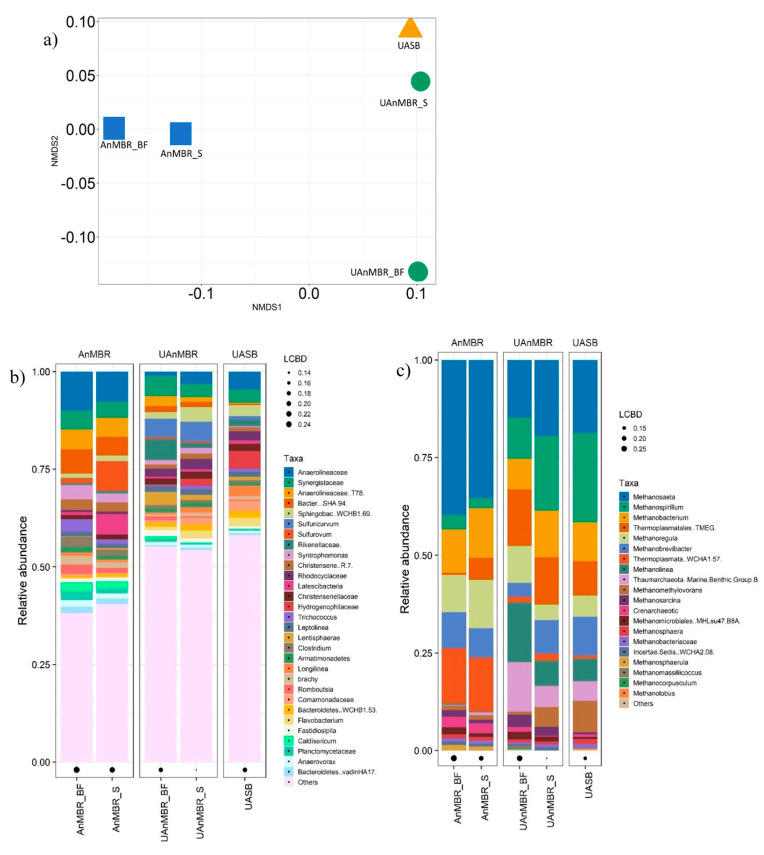
(**a**) Non-metric multidimensional scaling (NMDS) plot of reactor communities, (**b**) relative abundance of the 30 most abundant bacteria; (**c**) relative abundance of the 20 most abundant archaea. AnMBR_BF is the biofilm from AnMBR, AnMBR_S is the sludge from AnMBR, UAnMBR_BF is the biofilm from UAnMBR, and UASB represents sludge from the UASB.

The microbial communities in the sludge and biofilm of the AnMBR and UAnMBR were found to be different, but as expected, the UASB sludge community was similar to the sludge of the UAnMBR (Figure 4a). Both archaeal and bacterial communities were different in the AnMBR and UAnMBR (Figure 4 b,c).

The genus *Methanosaeta* was the main archaea (14.7–39.7% relative to total archaea) present in all three bioreactors followed by *Methanospirillum* (2.6–22.9%) and *Methanobacterium* (7.9–12.7%). In general, the genera *Methanosaeta*, *Methanoregula*, and *Thermoplasmatales* (*WCHA1*.57) had higher relative abundance in the AnMBR (both biofilm and sludge) than UAnMBR (biofilm and sludge) and UASB (sludge), while *Methanospirillum*, *Thermoplasmatales* (*TMEG*), and *Methanomethylovorans* tended to be richer in the UAnMBR and UASB compared to the AnMBR. The archaea *Thaumarchaeota* (*Marine benthic group B*) and *Methanolinea* were only present in the UASB and the UAnMBR (present in negligible abundance in AnMBR), with the UAnMBR biofilm showing the highest abundance. The relative abundance of *Methanosarcina*, *Crenarchaeotic*, *Methanomicrobiales (MHLsu47, B8A)*, and *Methanosphaera* were generally higher in the biofilms than in the sludge of both membrane bioreactors (AnMBR and UAnMBR).

In the case of bacteria (at the genus level), *Anaerolineaceae* (unclassified) (1.1–10% relative to total bacteria) and *Synergistaceae* (unclassified) (3.3–5.1%) were the most abundant taxa in all three bioreactors. The genus *Anaerolineaceae T78*, *Anaerolineaceae* (unclassified), *Bacteroidetes* (vadinHA17, SHA94), and *Sulfurovum* showed higher abundance in the AnMBR than UAnMBR and UASB. Generally, *Synergistaceae* (unclassified), *Anaerolineaceae* (T78), *Bacteroidetes* (*vadinHA17, SHA 94*), and *Clostridium* had higher relative abundance in the biofilms than the sludge. The genus *Sulfuricurvum*, *Longilinea*, *Lentisphaerae*, *Sphingobacteriaceae* (*WCHB1-69*), and *Leptolinea*, an amplicon sequence variant (ASV) from the family *Rikenellaceae* (unclassified) and *Hydrogenophilaceae* (unclassified) were the most abundant taxa in the UAnMBR and the UASB, but not in the AnMBR.

The relative abundance of *Anaerolineaceae* (unclassified) in the AnMBR biofilm and sludge was 10.0% and 7.7%, while in the UAnMBR biofilm and sludge, the proportion was 1.1% and 3.1%, respectively; for the UASB, this reached up to 4.6%. The relative abundance of the genus *Anaerolineaceae* (*T78*) was found in the AnMBR biofilm and sludge at 5.0% and 4.9%, while in the UAnMBR biofilm and sludge, its relative abundance was 2.6% and 1.3%, respectively, and this genus reached 2.7% in the UASB. The relative abundance of *Synergistaceae* (unclassified) in the AnMBR biofilm and sludge, UAnMBR sludge and biofilm, and UASB sludge was 4.8%, 4.1%, 5.1%, 3.3%, and 3.4%, respectively.

## 4. Discussion

The current study has investigated and compared AHL-mediated QS activity in the biofilm and the sludge of three different anaerobic bioreactors (AnMBR, UAnMBR, and UASB). To date, only a limited number of studies have briefly mentioned the status of AHL in relation to QS in anaerobic bioreactors [53,54], and so far, no study has focused specifically on the existence of AHL in the biofilms of such systems operating at low temperatures. This renders this study critical, as it could pave the way towards a better understanding of excessive biofilm formation on the surface of a membranes in MBR systems; this process leads inevitably to membrane fouling, which is the main cause of the increased operational and maintenance cost of membrane bioreactors [55]. Therefore, providing an insight into the status of AHL-mediated QS activity in these systems could eventually enable operators to devise new strategies for a fouling mitigation process that would reduce operating costs. This is critical for anaerobic bioreactors treating domestic wastewater, because the energy yields (as biogas) from such substrates are not sufficient to support their sustainable operation [6].

The importance of AHL C4-HSL, C6-HSL, 3-oxo-C6-HSL, C8-HSL, 3-oxo-C8-HSL, C10-HSL, 3-oxo-C10-HSL, C12-HSL, 3-oxo-C12-HSL, C14-HSL, and 3-oxo-C14-HSL has been reported recently in relation to different bacteria-mediated processes in mesophilic (37 °C) batch-fed anaerobic digesters, (organic loading rate of 1.5–2 kg COD m^−3^ day^−1^) fed with synthetic wastewater [30]. In that study, the total AHL concentrations were generally higher than those observed in the current study. However, the concentrations of all AHL present in a conventional UASB (37 °C, 2 kg COD m^−3^ day^−1^) were considerably lower (at 0–6 ng L^−1^) than those observed in the UASB, AnMBR, and UAnMBR of the current study (Figure 2), with C4-HSL being the highest (6 ng L^−1^) [30]. Additionally, C8-HSL, 3-oxo-C8-HSL, C10-HSL, and C12-HSL were not detectable in the conventional mesophilic UASB, but they were present in the UASB of the current study. A plausible reason could be the higher temperature (37 °C) of their experiment and use of synthetic wastewater (absence of non-acclimated cells). It may be significant that some AHL, namely C6-HSL, 3-oxo-C6-HSL, and 3-oxo-C8-HSL were not detected in the sludge or biofilm of the AnMBR, but they were found in the UASB and UAnMBR (both sludge and biofilm). Therefore, since these bioreactors both contained a more structured biomass in the form of granular sludge (or at least denser flocs), it is possible that the microbial communities in these UASB setups could have excreted these specific AHL as part of the granulation formation process that occurs naturally in upflow systems, which agrees with observations reported previously [53].

**Figure 5:**
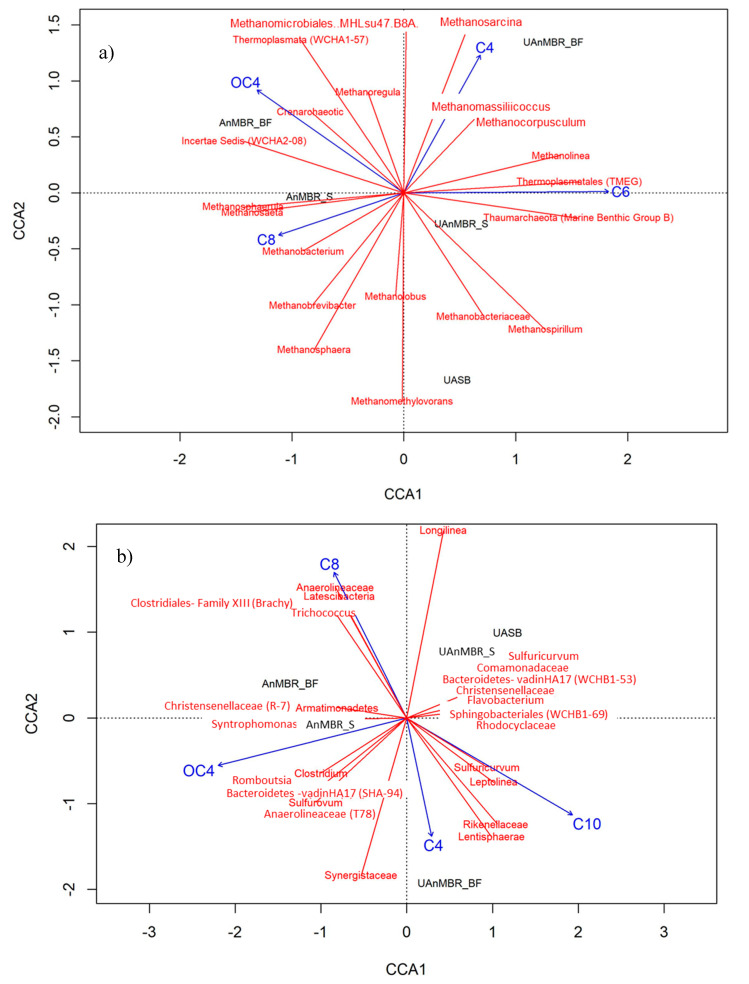
Canonical correspondence analysis (CCA) of acyl homoserine lactones (AHL) concentrations with (**a**) all archaea and (**b**) the 30 most abundant bacteria. AnMBR_BF is the biofilm from AnMBR, AnMBR_S is the sludge from AnMBR, UAnMBR_BF is the biofilm from UAnMBR, and UASB represents sludge from the UASB. AHL abbreviations are as follows; C4: C4-HSL; C6: C6-HSL; C8: C8-HSL; C10: C10-HSL; C12: C12-HSL; OC4: 3-oxo-C4-HSL; OC6: 3-oxo-C6-HSL; OC8: 3-oxo-C8-HSL; OC10: 3-oxo-C10-HSL; OC12: 3-oxo-C12-HSL.

**Figure 6:**
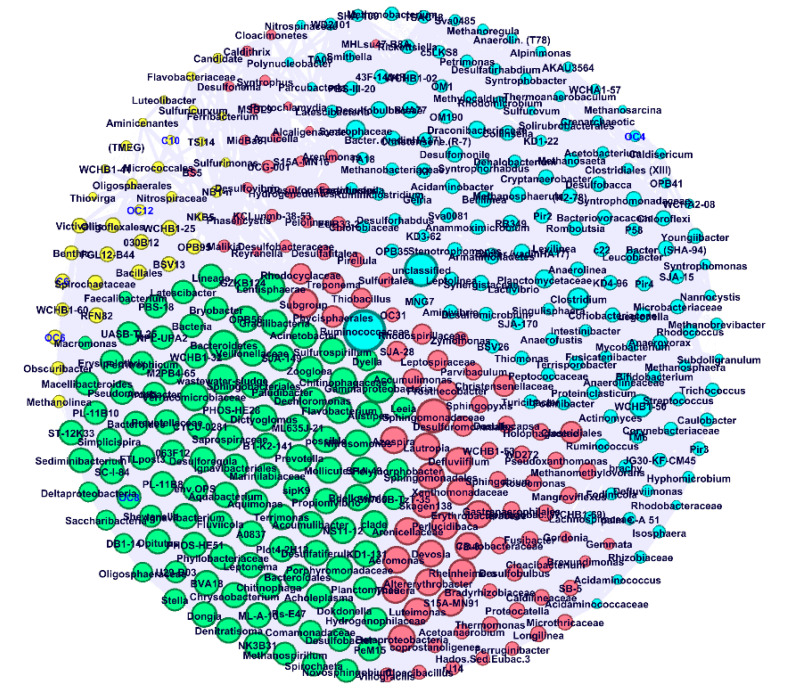
Co-occurrence network of biofilm and sludge of all reactors at genus level and AHL concentrations. The modularity of the nodes is differentiated by colors at genus level and AHL concentration. Only strong (Pearson’s R > 0.8) and significant (*p* < 0.05) correlation connections were included, and nodes were labeled for genus (black) and AHL concentration (blue).

Recently, Ma et al. [28] has reported the presence of only two kinds of AHL, C8-HSL (up to 250.0 ng L^−1^) and C10 HSL (up to 500.0 ng L^−1^), in mature anaerobic granules from a mesophilic digester fed with synthetic wastewater (OLR 4.0 kg COD m^−3^ day^−1^). The concentration of C10-HSL reported in their study [28] was 10-fold lower than the C10-HSL concentration found in our UASB and UAnMBR (both in sludge and biofilms) and comparable to the C10-HSL concentration in the AnMBR biofilm and sludge (1159.0 ng kg^−1^ and 184.4 ng L^−1^). Similarly, Ma et al. [29] also observed four AHL with maximum concentrations of C6-HSL (25 ng L^−1^), C8-HSL (420 ng L^−1^), C10-HSL (240 ng L^−1^), and C12-HSL (180 ng L^−1^) in a UASB treating industrial wastewater. The C6-HSL, C8-HSL, and C12-HSL concentrations were similar, whereas the C10-HSL concentration was 10-fold lower than that of the UASB sludge, UAnMBR biofilm, and UAnMBR sludge of the current study. Their maximum organic loading rate was 24.0 kg COD m^−3^ day^−1^ [29] (typical for an industrial wastewater), was 20-fold higher than the OLR of the current study which treated sewage (a low strength wastewater). This indicates that at higher OLR, the concentration of C10-HSL reduces; this suggests that C10-HSL is the main QS molecule used by the microbial community during low nutrient (low OLR) conditions and starvation stress. The use of AHL by microbial populations in upflow anaerobic bioreactors (UASB) has been reported previously to induce a k-strategy during starvation for reproduction and regulating cooperation [30]. The higher concentration (2-fold) of C10-HSL in UAnMBR biofilms as compared to UAnMBR sludge strengthens this argument. The biofilm would have had a higher microbial density than the sludge and hence greater competition for substrate between cells (or less substrate per unit of cells); consequently, higher concentrations of C10-HSL and other AHL were to be expected. Under substrate competition conditions, bacteria are known to produce more glucose-dominated EPS [56], which was the case observed in the current study based on the higher concentrations of polysaccharides in the biofilm (Figure 3). Furthermore, the canonical analysis linking community abundance with AHL concentrations showed that only bacteria were correlated with C10-HSL (Figure 5b and Figure 6).

**Figure 7:**
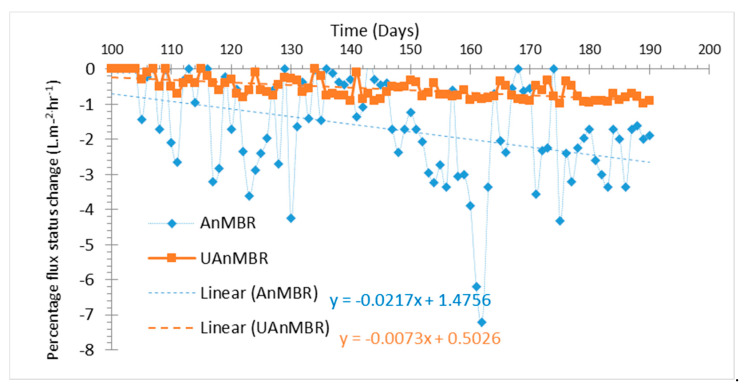
Percentage flux reduction in AnMBR and UAnMBR from the 100th (new membrane installed) to the 190th day.

Furthermore, C10-HSL concentrations in the AnMBR were lower than in the UAnMBR, despite the lower OLR of the former (0.10 kg COD m^−3^ day^−1^) compared to the latter (1.2 kg COD m^−3^ day^−1^). Regardless, the polysaccharides concentration was higher in the AnMBR biofilm, which was plausibly attributed to the higher concentration of 3-oxo-C4-HSL. The significant correlation (*p* < 0.05) of 3-oxo-C4-HSL with polysaccharides and proteins in EPS (Figure 3e) strengthens this argument. Furthermore, the C8-HSL concentration in the AnMBR biofilm was higher than UAnMBR biofilm (Figure 2a), which could be the reason for the higher level of polysaccharides. Similarly, higher fouling rates were observed in the AnMBR compared to UAnMBR. High OLR has been reported previously as a cause for higher fouling rates [57,58]. However, low COD removal efficiencies means that some of the organic matter remains untreated; this could potentially block the membrane pores and increase fouling rates [59] (i.e., particulate matter). This plausibility explains the reason behind the higher fouling rates in the AnMBR compared to UAnMBR (Figure 7). The food to microorganism ratio (F:M) has been reported as an important parameter controlling fouling. A lower F:M leads to starvation and bacteria excrete glucose-dominant SMP under such conditions [56], and this could lead to higher fouling rates. The same trend was observed in the AnMBR biofilm and sludge (higher concentrations of polysaccharide in SMP and LB-EPS) compared to the UAnMBR. The higher HRT in the AnMBR decreases the OLR and subsequently lowers F:M compared to the UAnMBR. In addition, the potential of granulation has been reported previously for alleviating fouling compared to suspended flocculant sludge [60]; this could have been another plausible reason related to the low fouling rates in UAnMBR. We propose that the higher AHL concentration in UAnMBR could have been most likely factor associated with the granulation that usually occurs in such upflow systems.

The non-metric multidimensional scaling showed that the microbial communities varied in the different reactors. Canonical correlation analysis (CCA) was carried out to find the correlation between the core communities (both archaea and bacteria) of the bioreactors (sludge and biofilm) and the AHL.

In the case of the archaea, C4-HSL correlated with *Methanosarcina*, *Methanomassiliicoccus*, and *Methanocorpusculum*; 3-oxo-C4-HSL correlated with *Crenarchaeotic* and *Incertae Sedis* (*WCHA2*-08); C6-HSL correlated with *Thermoplasmatales* (TMEG), *Thaumarchaeota (Marine Benthic Group.B)*, and *Methanolinea*; and C8-HSL correlated with *Methanobacterium*, *Methanosaeta*, and *Methanosphaera*. Previous studies [61,62] have reported *Methanosaeta harundinacea*, *Methanobacterium thermautotrophicus*, *Methanobacterium formicicum*, and *Methanosarcina mazei* as key archaeal genera and species linked to the production of AHL in anaerobic reactors; this observation shows some similarity with the current study (Figure 5a). However, further detailed genomic studies are required to investigate the genes involved in the production of AHL molecules in the archaea and whether there is any link between them and methane production, or any other step in the archaeal metabolism.

In the case of bacteria, C10-HSL correlated with *Sulfuricurvum*, *Leptolinea*, *Lentisphaerae,* and *Rikenellaceae*; C8-HSL correlated with *Anaerolineaceae* (family), *Latescibacteria*, *Trichococcus*, and *Clostridiales*-family XIII (*Brachy*); 3-oxo-C4-HSL correlated with *Syntrophomonas*, *Clostridium*, *Romboutsia, Bacteroidetes vadinHA17 (SHA-94)*, and *Christensenellaceae* (R-7).

In addition, a co-occurrence network revealed that long and medium-chain AHL (C6-HSL, 3-oxo-C6-HSL, C12-HSL, and 3-oxo-C12-HSL) correlated with the community that clustered in one of the modules (Yellow) (Figure 6). This indicates that the group of a particular community is closely associated with QS through these molecules.

A recent study reported *Romboutsia* was responsible for QS through the AI-2 molecule [63], but not through AHL. Similarly, a *Clostridium* species has been reported to mediate QS through peptides as their autoinducer QS molecules to make spores and excrete exotoxins [64,65]. Additionally, a few studies also reported correlations between AHL and taxa in the granular sludge of a UASB. Specifically, Ma reported that *Christensenellaceae* and *Longilinea* were correlated with C10-HSL and C8-HSL [28]. *Latescibacteria*, *Bacteroidetes vadinHA17*, *Syntrophomonas*, *Clostridium*, *Christensenellaceae* (*R-7*), and *Anaerolineaceae* (family) were reported as having a strong correlation with the AHL concentration in industrial anaerobic granules [29,53], which corroborates the observations in the current study. However, bacteria that correlate with AHL may not necessarily be producing these molecules themselves, but responding to them or adopting phenotypic behavior after sensing AHL in their environment [31,66]. So, single-strain studies in an anaerobic environment are required to further understand whether the QS is mediated or is a phenotypic behavior adopted in response to the presence of autoinducers. Bacteria from different environments have previously been separated and examined for QS activity i.e., flocculant sludge in sequencing batch bioreactors [53], sludge of aerobic MBR [23], activated sludge [67], bovine rumen [68], plants [69], but there is no published study that reports the isolation of these bacteria, especially from anaerobic systems, and tests them for QS activity mediated through AHL.

Furthermore, in the current study, the AHL status and the corresponding microbial community was investigated; however, the operational parameters were not exactly similar. Hence, it is not yet certain which variable affected AHL differentiation the most: the organic loading per gram of VS, the HRT, the membrane flux (LMH) and/or others. Further studies using similar operational parameters would be critical to understand the routes to AHL diversity.

Therefore, further research is required to establish the roles of different types of QS autoinducers in the biofilms and sludge of AnMBR/UAnMBR to allow the development of strategies to control fouling without affecting treatment and methane production efficiency, especially at relatively cold but sustainable (for domestic wastewater) temperatures.

## 5. Conclusions

The concentration of the total AHL was higher in the biofilm of the membranes of the anaerobic bioreactors compared to the sludge (in both AnMBR types). AHL concentration was found to be higher in the UAnMBR than in the AnMBR. However, the EPS concentration and fouling rates were lower in the UAnMBR than in AnMBR. This suggests that the different operational conditions of different reactor types do affect the microbial communities, and subsequently the AHL response, which is expressed in divergent AHL concentrations. This study reports that the C10-HSL, C4-HSL, 3-oxo-C4-HSL, and C8-HSL are the main AHL present in anaerobic reactors (with or without membranes), these molecules require special attention in future work to further understand their role in biofilm formation/fouling and granulation.

## Figures and Tables

**Table 1:** Operational parameters of upflow anaerobic membrane bioreactor (UAnMBR), membrane bioreactor AnMBR, and upflow anaerobic sludge blanket (UASB) reactor.

Reactor Type	AnMBR	UAnMBR	UASB
**Membrane type**	Hollow fiber, PVDF (0.1 µm)	Hollow fiber, PVDF (0.1 µm)	-
**Plant scale (volume)**	Lab scale (1 L)	Lab scale (1 L)	Lab scale (1 L)
**Wastewater feed**	Domestic sewage	Domestic sewage	Domestic sewage
**Influent COD (mg L^−1^)**	269.5 ± 22.7	269.5 ± 22.7	269.5 ± 22.7
**Organic loading rate (kgCOD.m^−3^ d^−1^)**	0.108 ± 0.011	1.2 ± 0.17	1.2 ± 0.17
**pH**	6.7–7.2	6.7–7.2	6.7–7.2
**Temperature (°C)**	15	15	15
**HRT (Hours)**	60	7	7
**SRT (days)**	60	60	60
**Flux (L m^−2^ hr^−1^)**	0.75	2.18	-
